# Linking the plasma proteome to genetics in individuals from continental Africa provides insights into type 2 diabetes pathogenesis

**DOI:** 10.1038/s41588-025-02421-w

**Published:** 2026-01-08

**Authors:** Opeyemi Soremekun, Young-Chan Park, Mauro Tutino, Ana Luiza Arruda, Allan Kalungi, N. William Rayner, Moffat Nyirenda, Segun Fatumo, Eleftheria Zeggini

**Affiliations:** 1 Institute of Translational Genomics, Computational Health Center, Helmholtz Zentrum München – German Research Center for Environmental Health, Neuherberg, Germany; 2 Molecular Bio-computation and Drug Design Laboratory, School of Health Sciences, University of KwaZulu-Natal, Durban, South Africa; 3 Medical Research Council, Uganda Virus Research Institute and London School of Hygiene and Tropical Medicine (MRC/UVRI &LSHTM), Entebbe, Uganda; 4 Munich School for Data Science, Helmholtz Munich, Neuherberg, Germany; 5 Technical University of Munich, School of Medicine and Health, Graduate School of Experimental Medicine, Munich, Germany; 6 Department of Non-communicable Disease Epidemiology, London School of Hygiene and Tropical Medicine, London, UK; 7 Department of Medical Biochemistry, College of Health Sciences, Makerere University, Kampala, Uganda; 8 Precision Healthcare University Research Institute Queen Mary University of London, London, UK; 9 Technical University of Munich (TUM), TUM University Hospital, TUM School of Medicine and Health, Munich, Germany

**Keywords:** Metabolic disorders, Diseases

## Abstract

Individuals of African ancestry remain largely underrepresented in genetic and proteomic studies. Here we measure the levels of 2,873 proteins in plasma samples from 163 individuals with type 2 diabetes (T2D) or prediabetes and 362 normoglycemic controls from the Ugandan population. We identify 88 differentially expressed proteins between the two groups. We link genome-wide data to protein expression levels and construct a protein quantitative trait locus (pQTL) map for this population. We identify 399 independent associations with 346 (86.7%) *cis*-pQTLs and 53 (13.3%) *trans*-pQTLs; 16.7% of the *cis*-pQTLs and all of the *trans*-pQTLs have not been previously reported in individuals of African ancestry. Of these, 37 pQTLs have not been previously reported in any population. We find evidence for colocalization between a pQTL and T2D genetic risk. Our findings reveal proteins causally implicated in the pathogenesis of T2D, which may be leveraged for personalized medicine tailored to individuals of African ancestry.

## Abstract (web summary)

This study uses a high-dimensional proteomics panel to explore protein-level genetic associations with type 2 diabetes in a Ugandan cohort.

## Main

Type 2 diabetes (T2D) is becoming a major public health concern in Africa, congruent with the complex interplay of genetic, environmental and socioeconomic factors^1–3^. According to the International Diabetes Federation, it is predicted that, globally, people with T2D will rise by 51%, reaching 700.2 million by 2045 from 463 million in 2019^4^. A substantial increase of 143% is anticipated in Africa, with numbers expected to rise from 19.4 million in 2019 to 47.1 million in 2045^4^. Hemoglobin A1c (HbA1c), also known as glycated hemoglobin^5^, provides an estimate of the blood sugar level over a period of 2–3 months by measuring the percentage of hemoglobin with attached glucose^6,7^. An HbA1c level of 6.5% or higher on two separate tests typically indicates diabetes. Levels between 5.7% and 6.4% suggest prediabetes, and values below 5.7% are considered normal^8^. Combining proteomic and genomic data for blood-based protein quantitative trait loci (pQTLs) has identified hundreds of associations between genetic variants and protein levels^9–13^. A fraction of individuals with African ancestry in the diaspora has been studied in proteomics studies to date^12,14^, with continental Africans largely underrepresented.

To address this, we measured 2,873 proteins using the Olink PEA Explore assay in the plasma samples of 163 individuals with prediabetes or T2D (cases) (defined as HbA1c > 5.7%) and 362 normoglycemic controls (defined as HbA1c < 5.7%) (Table 1) from a subset of the Uganda Genome resource, hereafter referred to as Uganda Genome Resource Proteomics Data (UGR-PD). We performed differential protein expression analysis between the two groups and carried out proteomic genetic association analysis to identify sequence variants influencing protein levels. We subsequently examined the role of the identified pQTLs in T2D using colocalization and Mendelian randomization (MR) analyses.

**Table 1:** Clinical characteristics of the study participants

	Cases	Controls
Number of participants, *n* (%)	163 (31.05)	362 (68.95)
Age (years), mean ± s.d.	49.82 ± 18.5	50.39 ± 17.76
Male, *n* (%)	47 (28.83)	139 (38.40)
Female, *n* (%)	116 (71.17)	223 (61.60)
BMI, kg m^−2^	23.4	22.1
HbA1c, %	6.46 ± 1.24	5.13 ± 0.48

BMI, body mass index.

**Fig. 1:**
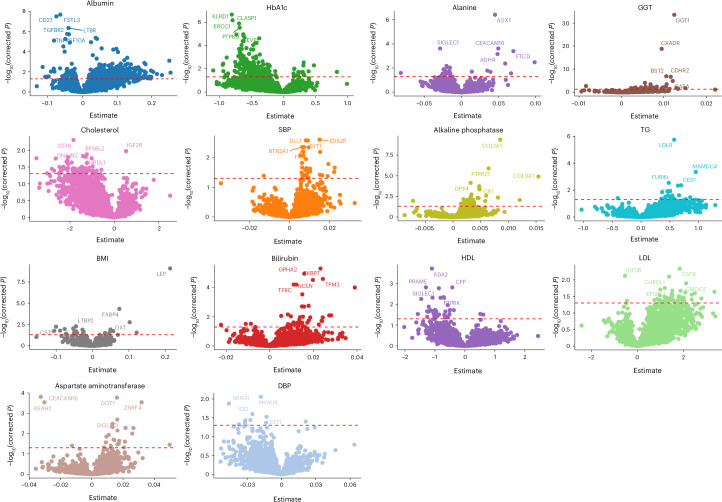
Association of protein levels with clinical traits. The *y* axis represents the association’s FDR-adjusted −log_10_(*P*); the *x* axis of each plot represents the effect size estimated using linear regression. The horizontal red dashed line indicates the multiple testing adjusted significance threshold with associations above the line considered statistically significant. GGT, gamma-glutamyl transferase; SBP, systolic blood pressure.

First, we studied the association between protein levels and cardiometabolic traits measured in the UGR-PD (Supplementary Table 1). A total of 208 proteins were associated with HbA1c, 42 with high-density lipoprotein (HDL) and 46 with low-density lipoprotein (LDL) at a false discovery rate (FDR) of 5% (Fig. 1). Some of the associations, such as ERCC1 found to be associated with HbA1c (*P*_adj_ = 6.77 × 10^−7^) and HDL (*P*_adj_ = 1.91 × 10^−2^), have been shown to affect glucose intolerance in a progeroid-deficient animal model causing an autoinflammatory response that leads to fat loss and insulin resistance^15^.

**Fig. 2:**
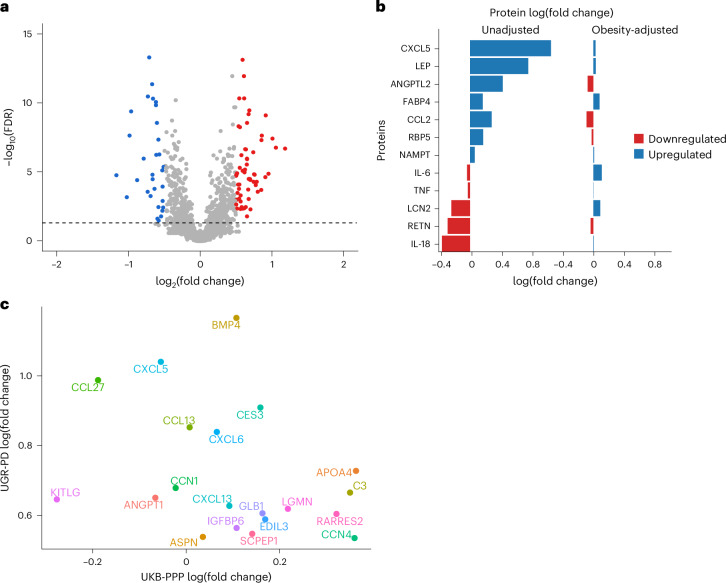
Proteomic profiling identifies differentially expressed proteins linked to type 2 diabetes. **a**, Volcano plot showing DEPs, with significantly overexpressed proteins annotated in red and downregulated proteins in blue, using a linear model implemented in limma. The black horizontal dashed line represents the −log_10_(FDR) cutoff corresponding to a 5% false discovery rate. **b**, Comparison of cases and controls with regard to adipokines and other proteins that are biomarkers of obesity and central adiposity before and after adjusting for obesity. The log(fold change), a measure of protein expression changes between patients with T2D and controls, was calculated as the base-2 logarithm of the ratio of the mean expression in patients with T2D to the mean expression in controls. **c**, Scatter plot of the comparison of the top significant DEPs with UGR-PD on the *y* axis and UKB-PPP on the *x* axis.

Next, we sought to identify differentially expressed protein (DEP) levels between cases and controls. DEPs were defined based on a twofold change (log_2_(fold change) > 0.5) in expression levels at an FDR of 5%. This led to the identification of 88 DEPs. Among these, 57 were significantly upregulated, with log_2_ fold changes ranging from 0.50 to 1.18, while 31 proteins were downregulated with log_2_ fold changes between −0.51 and −1.17 (Fig. 2a and Supplementary Table 2). EGF-like repeats and discoidin I-like domains 3 (EDIL3), associated with processes such as cell adhesion, migration and vascular development, showed the most significant upregulation with *P*_adj_ 1.2 × 10^−13^. EDIL3 is differentially expressed in the adipose tissue of insulin-resistant and insulin-sensitive individuals^16,17^, and is involved in angiogenesis^18–20^. Impaired angiogenesis has been implicated in the progression of diabetic retinopathy and nephropathy^21,22^. The DEPs were primarily enriched in Gene Ontology terms such as chemokine receptor binding and chemokine and cytokine activity (Supplementary Table 3). We further compared cases and controls with regard to adipokines, biomarkers of obesity and proteins linked to pancreatic function before and after adjusting for obesity to disentangle obesity-driven signals from those independently associated with diseases status (Fig. 2b). In cases of the unadjusted model, leptin (LEP) was significantly upregulated compared to controls (log(fold change) = 0.759, *P*_adj_ = 1.62 × 10^−5^). C-X-C motif chemokine ligand 5 (CXCL5) showed the highest upregulation in cases (log(fold change) = 1.056, *P*_adj_ = 1.76 × 10^−7^). Resistin and interleukin-18 were significantly downregulated in cases compared to controls (log(fold change) *P*_adj_ = −0.292, 8.51 × 10^−3^ and −0.367, and 5.89 × 10^−4^, respectively). Additionally, angiopoietin-like protein 2 was elevated in cases (log(fold change) = 0.426, *P*_adj_ = 0.00153), while inflammatory markers such as tumor necrosis factor and interleukin-6 showed nonsignificant expression level differences between cases and controls. However, upon adjusting for obesity, CXCL5 and LEP were attenuated indicating that their expressions may be mediated by obesity (Fig. 2b).

The comparison of significant DEPs in UGR-PD with the same set of proteins in the UK Biobank Pharma Proteomics Project (UKB-PPP) using the T2D definition described in ref. ^23^ (*n*_cases (T2D)_ = 2,461 and *n*_controls_ = 50,553) showed some population-specific differences (log(fold change)). For instance, proteins such as apolipoprotein F (APOF), tumor necrosis factor superfamily member 12 and lipoprotein lipase (LPL) are significantly upregulated in patients with T2D compared to controls in the UGR-PD but not in the UKB-PPP. lysophosphatidylcholine acyltransferase 2 and interleukin-8 are more strongly downregulated in patients with T2D compared to controls in the UGR-PD. Proteins such as prolylcarboxypeptidase, LEP, EDIL3 and apolipoprotein A-IV (APOA4) showed the same trend of expression between patients with T2D and controls in the two populations (Fig. 2c).

Among the significant DEPs in the UGR-PD, eight have T2D-associated genome-wide association study (GWAS) hits within 40 kb (Table 2), although none of the significant DEPs showed evidence of colocalization with T2D. The association of these proteins with T2D and the nearby GWAS signals strengthens the hypothesis that these proteins could have a causal or mediatory role in the pathophysiology of T2D in this population.

**Table 2:** Significant DEPs with a T2D GWAS hit within 40 kb of the transcription site of the gene encoding the protein

Protein	GWAS hit	GWAS hit (reported gene)	Distance (kb)
LEP	7:128223242	LEP,MIR129-1	19
CCN4	8:133184606	TG,CCN4	6
FARSA	19:12927601	FARSA	5
NMI	2:151310003	TNFAIP6,NMI	40
APOA4	11:116809702	LNC-RHL1,APOA5	11
IGFBP6	12:53083566	SPRYD3,IGFBP6	14
APOF	12:56347444	STAT2	13
LPL	8:19922799	LPL	21

After quality control, we undertook pQTL analysis with up to 15.8 million imputed variants with a minor allele frequency (MAF) > 0.05 for 2,873 proteins. We identified 399 independent associations after multiple testing correction at *P* value thresholds of *P* < 1.46 × 10^−6^ and *P* < 2.2×10^−10^ for *cis-* and *trans*-pQTLs, respectively (Supplementary Table 4). We identified 346 (86.7%) *cis-*pQTLs and 53 (13.3%) *trans-*pQTLs. Seven proteins had both *cis*-pQTLs and *trans-*pQTLs. We also identified four *trans*-pQTLs located within a pleiotropic locus.

To determine the uniqueness of the pQTLs identified in the UGR-PD, we compared them against the pQTLs of 47 genome-wide pQTL studies (Supplementary Table 5). We identified six independent *cis*-pQTLs and 31 independent *trans*-pQTLs that were not previously reported in any population (Supplementary Table 6), and 362 pQTLs reported in prior studies (Supplementary Table 7). We compared our pQTL findings against the African ancestry data of the UKB-PPP and found that 16.7% (58 of 346) of the discovered *cis*-pQTLs and all *trans*-pQTLs have not been reported previously (Supplementary Table 8). We tested the conditionally independent UGR-PD pQTLs for replication in the UKB-PPP. Of the 399 pQTLs, we were able to test 392 in the UKB-PPP data. Of these, 303 replicated at *P* ≤ 1.2 × 10^−4^ (Bonferroni-corrected threshold) and 270 also had the same effect estimate direction (Supplementary Table 9).

We examined the relevance of the previously identified pQTLs with T2D and associated risk factors, such as lipid traits, blood pressure and cardiovascular disease, by cross-referencing with the GWAS Catalog and ref. ^24^. Of the 362 previously identified pQTLs (Supplementary Table 7), six were associated with T2D or T2D-related traits (Supplementary Table 10).

**Fig. 3:**
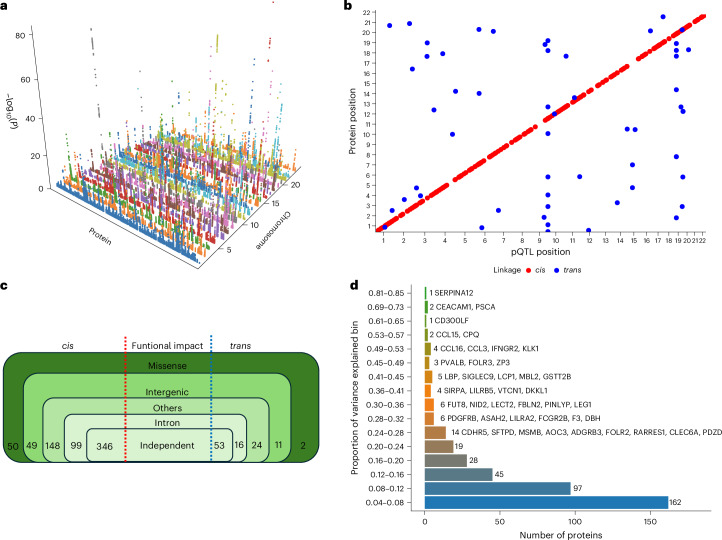
Three-dimensional Manhattan plot of identified *cis*-pQTLs. **a**, Proteins are shown on the *x* axis, chromosome location is shown on the *y* axis and the −log_10_(*P*) of each association is shown on the *z* axis. **b**, Scatter plot of pQTL variant location against the location of the gene encoding the target protein. Each dot represents an independent variant. *cis*-pQTLs are colored in red, while *trans*-pQTLs are colored in blue. A multiple testing correction threshold was used for both *cis* and *trans*-pQTLs. **c**, Summary of the identified pQTLs showing their functional consequences. **d**, Proportion of variance explained by the conditionally independent pQTLs categorized into bins.

One hundred and fifty-one identified pQTLs overlapped or fell within a 500-kb window of T2D-associated GWAS variants (Supplementary Table 11). Only one of these pQTLs (rs6075339) colocalized with a T2D signal. rs901886 (ICAM5) located on chromosome 9 overlapped with multiple T2D-associated variants, including rs74956615 and rs34536443, which have been implicated in immune regulation and inflammation^25,26^, processes known to contribute to T2D pathophysiology. rs62068711 (DPEP1) on chromosome 16 also overlaps with rs12920022, a variant previously linked to T2D risk^27^, suggesting a potential role of dipeptidase-related pathways in glucose metabolism. Furthermore, a pleiotropic pQTL, rs532436, identified near SELE, IL-7R and ALPI in our study is also associated with a GWAS hit (rs529565) for ABO protein levels^28^. The association of rs532436 with multiple proteins (for example, ABO, SELE, IL-7R) suggests that this variant may affect upstream regulatory mechanisms (for example, transcription factor binding, chromatin accessibility) influencing the expression of multiple genes (Fig. 3).

**Fig. 4:**
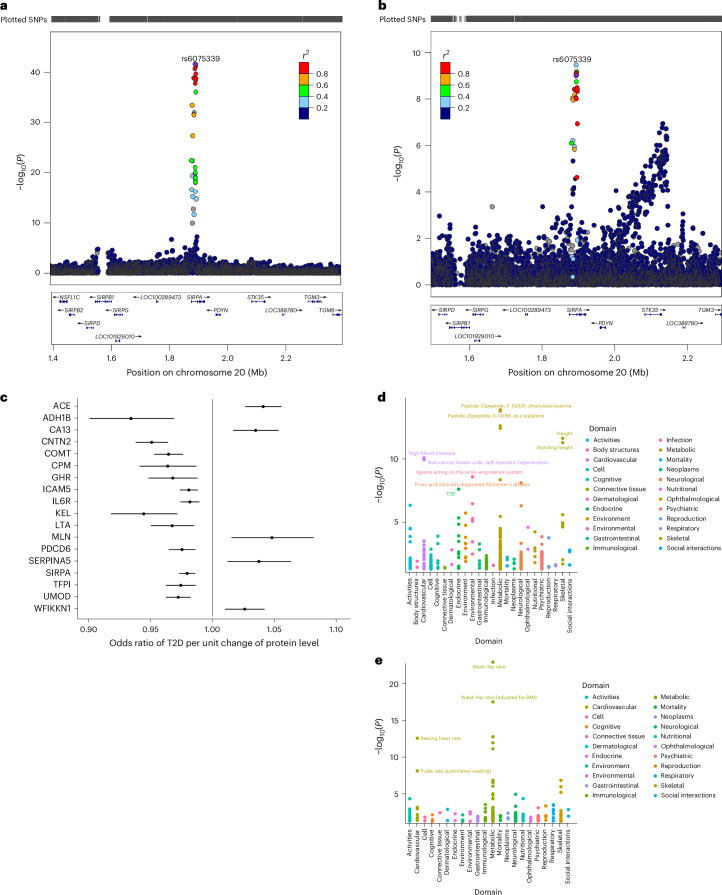
LocusZoom plots of the colocalizing SIRPα pQTL and T2D risk variant. **a**,**b**, LocusZoom plots of the colocalizing SIRPα pQTL (**a**) and T2D risk variant (**b**). Top: T2D GWAS *P* values. Bottom: pQTL *P* values for the same region. **c**, MR forest plot for proteins causally associated with T2D. The effect estimates represent the odd ratio of T2D per unit change of protein level and the error bars represent the 95% confidence intervals around the estimated effects. These were estimated using a Wald ratio estimate. **d**,**e**, PheWAS plots for TFP1 (**d**) and ACE (**e**). SNP, single-nucleotide polymorphism.

Next, we performed colocalization analysis to determine the shared risk variants between pQTLs and T2D using a large multi-ancestry GWAS^29^. We found one colocalizing signal with strong evidence for a shared T2D risk variant. Specifically, we observed a posterior probability (PP4 = 95.5%) for colocalization between a T2D-associated variant and a pQTL (rs6075339) regulating the expression of the signal regulatory protein alpha (SIRPα) protein (Fig. 4a,b). Genetic studies have implicated SIRP signaling in diabetes pathogenesis. For example, a single-nucleotide polymorphism in human SIRPγ, encoding a SIRP family receptor that also binds CD47, was associated with type 1 diabetes^30^.

We undertook an MR analysis to examine the causal relationship between the identified *cis*-pQTLs and T2D. We found 18 proteins to be causally associated with T2D. Our MR results showed that genetically increased angiotensin-converting enzyme (ACE), CA13, MLN, SERPINA5 and WFIKKN1 levels were associated with an increased risk of T2D. Proteins such as ADH1B, CNTN2, COMT, CPM, GHR, ICAM5 and ILR6 showed a protective effect on T2D risk (Fig. 4c and Supplementary Table 12). ACE is an essential component of the renin–angiotensin system and it has a crucial role in the development of insulin resistance^31^. By increasing insulin sensitivity and decreasing inflammation, ACE inhibitors, which are frequently used to treat hypertension, have been demonstrated in clinical studies and meta-analyses to lower the incidence of new-onset T2D in people at high risk^32^. the *COMT* variant rs4680 is associated with lower HbA1c and protection from T2D^33^. This corroborates our MR findings where the COMT pQTL rs4680 showed a protective effect against T2D. While no other significant pQTLs identified through MR were directly associated with T2D, several proteins (TFPI, LTA, GHR and ADH1B) encoded by genes within which these pQTLs reside have been linked to T2D or T2D-related traits (Supplementary Table 13).

In line with its established function in blood pressure regulation, the pQTL rs4363 showed significant associations with cardiovascular traits in the phenome-wide association study (PheWAS), such as high blood pressure and hypertension. Furthermore, its associations with Alzheimer’s disease (neurological domain) and T2D (metabolic domain) indicate wider in metabolic and neurodegenerative processes. It also showed some significant associations with anthropometric traits, such as height and standing height. rs3213739 exhibited significant associations with the waist–hip ratio (anthropometric domain) and the resting heart rate and pulse rate (cardiovascular domain), highlighting its role in body composition and metabolism (Fig.  4d,e and Supplementary Table 14).

Lastly, we assembled a list of 1,804 postulated effector genes for T2D from nine GWAS studies. If a gene coding for any of the proteins associated with the identified pQTLs in our study was found in the curated list, we defined such gene/protein as reported; if not, we classified them as previously unresolved. We identified 320 proteins previously unresolved as potentially linked to effector genes for T2D based on these GWAS signals (Supplementary Table 15).

Our work takes a first step toward addressing the underrepresentation of continental African individuals in genetics and proteomics studies. Thus, we were able to delineate the molecular landscape of 2,873 unique proteins in a context that might be pivotal to understanding drivers of T2D pathophysiology, identified 58 African-ancestry-specific *cis*-pQTLs that have not been reported previously and identified 18 proteins that are causally associated with T2D. The generalizability of these findings may be limited to the continent because the population was drawn from a single demographic group within Africa. Hence, there is a need to include more ancestrally diverse populations in future studies.

In this study, we used the Olink targeted proteomic assay, which has some limitations; for example, only a subset of the full proteome is studied and the affinity of aptamers may be affected by missense variants. While HbA1c is a highly standardized and accurate test with lower intraindividual variability compared to fasting glucose, in individuals of African ancestry, using HbA1c as a blood sugar level indicator may not provide the full spectrum of the metabolic conditions associated with T2D because of the prevalence of hemoglobinopathies, such as glucose-6-phosphate dehydrogenase (G6PD) deficiency. In individuals with G6PD deficiency, there is increased susceptibility to hemolysis, which may lead to reduced HbA1c levels potentially leading to missed T2D diagnosis^34,35^.

The DEP analysis of adipokines and metabolic proteins between cases and controls revealed differences in the role these proteins have in obesity, inflammation and pancreatic function. LEP was significantly upregulated in cases, which is consistent with its known association with adiposity and metabolic regulation^36^. Previous studies linked circulating LEP levels with insulin resistance and T2D development^37^; experimental models suggest that it may influence Beta cell function and glucose metabolism^38,39^.

Population-specific differences in protein expression were observed when DEPs were compared between the UGR-PD and UKB-PPP cohorts. Some proteins were upregulated in patients with T2D compared to controls in one cohort but not in the other. In comparison, other proteins were downregulated in one cohort but upregulated in the other. These differences suggest that factors beyond disease status may influence variation in protein expression. Ancestral genetic variation is one potential explanation, as genetic diversity affects gene regulation and metabolic pathways^40^. Additionally, environmental factors, including diet, lifestyle and exposure to infections, may contribute to disparities in protein expression profiles. Lastly, variations in T2D disease progression, comorbidities or medication use across the two cohorts could also have a role. Some significantly expressed DEPs had a T2D GWAS hit within a 500-kb window. However, none colocalized with T2D. The finding provides evidence that disease risk may be influenced by genetic variants close to T2D-associated proteins via protein-mediated pathways. Proteins like LEP, LPL, EIF5A and CCL25 have several GWAS hits within ±500 kb of them, which shows that these proteins may mediate genetic predisposition to T2D.

Some of the identified pQTLs were associated with T2D or relevant to T2D via association with other cardiometabolic traits, including lipid and blood pressure traits. Previous studies found rs532436 and rs505922 to be associated with T2D, HDL cholesterol levels, triglycerides (TGs) and diastolic blood pressure (DBP) ^41–43^ across diverse ancestral populations. In addition, rs77924615 has been linked to cardiovascular disease and blood pressure traits^44,45^, supporting its potential contribution to metabolic syndrome, a key risk factor for T2D. The association of rs10460181, rs2455069 and rs12721054 with lipid traits^46–48^ corroborate previous findings that lipid dysregulation has a vital role in developing insulin resistance and T2D^49,50^. According to the MR results, the COMT pQTL rs4680 had a protective effect against T2D. This is consistent with a study conducted in the Women’s Genome Health Study, which found that the high-activity G-allele of rs4680 was linked to lower HbA1c levels and a slight decrease in the risk of T2D in women of European ancestry^33^.

In conclusion, the associations and causally associated proteins identified offer promising avenues for developing targeted therapies and personalized treatment strategies for T2D, contributing to improved management and prevention of this global health challenge. Our findings demonstrate the utility and discovery opportunities afforded by including individuals of African ancestry in large-scale proteomic studies.

## Methods

### Ethics

The study was approved by the Uganda Virus Research Institute Research and Ethics Committee (UVRI REC no. GC/127/907) and the Uganda National Council for Science and Technology (no. UNCST HS2527ES).

### Study population

Participants were selected from the UGR, a subset of the General Population Cohort (GPC). As described previously^51,52^, the GPC is a population-based cohort of over 22,000 people from 25 nearby communities in the remote Southwest Ugandan sub-county of Kyamulibwa, which is a part of the Kalungu district. We selected 528 samples from the UGR-PD based on age, sex and HbA1c. After hemolysis of anticoagulated whole blood, the concentrations of total hemoglobin and HbA1c were measured using turbidimetric inhibition immunoassay quantitative hemoglobin Alc Gen^51^. In addition to the genotype quality control described in ref. ^51^, we used a Hardy–Weinberg *P* < 1 × 10^−6^.

### Association with clinical characteristics

We used linear regression to determine the association between protein levels and systolic blood pressure, DBP, alanine, albumin, alkaline phosphatase, aspartate aminotransferase, bilirubin, cholesterol, gamma-glutamyl transferase, HDL, LDL, TGs and hemoglobin A1c. All *P* values were FDR-corrected.

### DEPs and functional enrichment

We determined DEPs between cases and controls using limma^53^; we used a Benjamini–Hochberg FDR for multiple testing^54^. DEPs are defined as proteins with an FDR < 5% and a fold change greater than 0.5 (log_2_(fold change) > 0.5). To better understand the functional impact of the proteins, we used the enrichr tools from clusterProfiler^55^.

### Proteomics quality control

The Olink’s proximity extension assay technology^56^ was used to measure the plasma level of 2,978 proteins in 528 samples across eight Olink panels. The levels of protein expression were measured logarithmically as Normalized Protein eXpression units. We adjusted all phenotypes using a linear regression for age, sex, plate number and sample collection season, followed by an inverse-normal transformation of the residuals. During the quality control process, we excluded one sample because the PCR plate well was empty; an additional two samples were further excluded because of a missingness greater than 40%. For assay quality control, 40 assays were excluded because they did not have Normalized Protein eXpression values. Additionally, we excluded 31 assays that had a fraction of assay warning greater than 15%. No assay was excluded because of limit of detection. In all, 525 samples and 2,873 assays remained after quality control and were subsequently used for further analysis.

### Single-point association

Covariates such as sex, age, plate and mean protein expression per sample were regressed using R’s LM function. Residuals were then translated into *z*-scores and used for the association analysis. We used the single-point-analysis-pipeline v.0.0.2 (dev branch) (https://github.com/hmgu-itg/single-point-analysis-pipeline/tree/dev) to perform the association analysis for single-nucleotide polymorphisms with a MAF > 0.05. GCTA v.1.93.2 beta was used to conduct a mixed linear model association analysis; the genetic relationship matrix function within the GCTA software was used to estimate the genetic relationships among individuals. We then used GCTA-COJO, designed for approximate conditional and joint stepwise model selection, to identify independent associated variants at each locus.

### Significance threshold

The confidence interval significant threshold was determined by multiplying the Bayes factors by the number of proteins tested; values over 1 were capped at 1. The Bayes factor was estimated using eigenMT^57^. eigenMT calculates *M*_eff_ as the number of ranked eigenvalues from the adjusted genotype correlation matrix needed to account for 99% of the detected genotype variability. Subsequently, the corrected *P* values were adjusted for multiple testing by applying the FDR method. *Q* values were then calculated using the qvalue package, allowing for the identification of a subset of significant associations based on a *q* < 0.05. Finally, the *cis* threshold for significance in the pQTL analysis was determined by averaging the smallest nonsignificant *P* value and the largest significant *P* value. This method resulted in a *cis* *P* = 1.462 × 10^−6^. The *trans* threshold was calculated based on the effective number of variants (*N*_eff_) and the number of protein traits (*M*_eff_). The *N*_eff_ was derived by performing linkage disequilibrium pruning with the indep 500 5 0.2 parameters in Plink v.1.9^58^. This resulted in an *N*_eff_ of 452,593 unique variants. The *M*_eff_ was calculated using the *M*_eff_ function and Gao method in the poolr R package^59^. The *trans* *P* value threshold is 2.227 × 10^−10^. Variants within 1 megabase (Mb) upstream or downstream of the encoding genes are referred to as *cis*-pQTLs, while *trans*-pQTLs are those found beyond 1 Mb relative to the encoding gene. Ensembl’s Variant Effect Predictor was used to determine the functional impact of the variants.

### Comparison of pQTLs to prior published data

To determine the uniqueness of our pQTLs, we used an in-house-built database of previously identified signals of 46 genome-wide pQTL studies, including the UKB-PPP^12^. We evaluated novelty by identifying new loci and new variants. New loci were defined as those with no published variants within ±1 Mb of our variants. For variants at known loci, we checked their rsIDs against those previously reported. Variants with no prior matches were further conditioned (gcta-cojo-cond) in the context of other known variants at that locus. These were classified as new if the significance of their association *P* value (*cis*-pQTL: *P* < 1.462 × 10^−6^ and *trans*-pQTL *P* < 2.227 × 10^−10^) persisted even after adjusting for other known variants.

### Colocalization analysis

We performed Bayesian-based colocalization analysis using the Coloc.fast function (https://github.com/tobyjohnson/gtx) between our pQTL signals and multi-ancestry T2D GWAS summary statistics^29^ from the DIAGRAM database. To assume shared genetics, we used default priors and a posterior probability of PP.H4 ≥ 0.8 (ref. ^60^). To increase statistical power and strengthen the robustness of our findings, a multi-ancestry GWAS (*n* = 2,535,601) was selected for the colocalization analysis rather than the largest African-specific meta-analysis (*n* = 154,160). The much larger sample sizes available in the multi-ancestry GWAS data facilitate higher resolution for signal localization and enhance the capacity to detect genetic associations.

### MR

To identify putative causal effects, we performed a two-sample MR analysis using the *cis*-pQTL data in the UGR-PD as exposure and the multi-ancestry T2D GWAS meta-analysis^29^ as the outcome. The analyses were conducted using the TwoSampleMR^61^. We used the previously defined independent *cis*-pQTLs as genetic instrumental variables and considered only those with an *F*-statistic greater than ten. As all proteins had at most one independent *cis*-pQTL, we applied the Wald ratio estimate. The use of single instrumental variables limits the sensitivity analyses for assessing MR assumptions. Therefore, we assessed consistency in the direction of effects using the African T2D GWAS meta-analysis^29^. We chose the multi-ancestry T2D GWAS meta-analysis for the primary results to maximize statistical power, acknowledging that the population structure of the African T2D GWAS meta-analysis is also not entirely homogeneous with the UGR-PD. Moreover, we corroborated our findings with a colocalization analysis. However, differences in linkage disequilibrium structures between the pQTLs and T2D GWAS data reduced the power to detect colocalizing signals.

### PheWAS

The PheWAS module of the GWAS Atlas^62^, a comprehensive database that integrates the findings of GWAS across several phenotypes and traits, was used to carry out the PheWAS. The analysis aimed to methodically assess a protein’s association with several phenotypes and traits. To account for the large number of tests, the module performs multiple testing corrections and organizes phenotypes into specified trait groups (such as metabolic, cardiovascular and immunological). A Bonferroni-corrected *P* = 1.05 × 10^−5^ was used to determine whether an association was significant.

### Identification of effector genes

To find putative effector genes for T2D, we compiled effector genes associated with the T2D GWAS. This dataset was curated from nine papers published in the Type 2 Diabetes Knowledge Portal, resulting in a collection of 1,804 distinct effector genes. For classification purposes, proteins that were documented in our curated list were labeled ‘reported’. Those not found on the list were classified as ‘unresolved’.

### Reporting summary

Further information on research design is available in the Nature Portfolio Reporting Summary linked to this article.

## Online content

Any methods, additional references, Nature Portfolio reporting summaries, source data, extended data, supplementary information, acknowledgements, peer review information; details of author contributions and competing interests; and statements of data and code availability are available at 10.1038/s41588-025-02421-w.

## Supplementary information

Reporting Summary

Supplementary Tables Supplementary Tables 1–16.

## Data Availability

The summary statistics for the significant pQTLs, and the results from the colocalization, are provided in Supplementary Tables 1–16. The full pQTL summary statistics are available for download from the GWAS Catalog (https://www.ebi.ac.uk/gwas/) under accession nos. GCST90648168–GCST90651039. Accession codes for the summary statistics of each protein are also provided in Supplementary Table 16.

## Code availability

The analyses were performed using publicly available software.

## References

[1] Tremblay, J. & Hamet, P. Environmental and genetic contributions to diabetes. *Metabolism* **100**, 153952 (2019). 10.1016/j.metabol.2019.153952 31610851

[2] Tekola-Ayele, F., Adeyemo, A. A. & Rotimi, C. N. Genetic epidemiology of type 2 diabetes and cardiovascular diseases in Africa. *Prog. Cardiovasc. Dis.* **56**, 251–260 (2013). 24267432 10.1016/j.pcad.2013.09.013 PMC3840391

[3] Motala, A. A., Mbanya, J. C., Ramaiya, K., Pirie, F. J. & Ekoru, K. Type 2 diabetes mellitus in sub-Saharan Africa: challenges and opportunities. *Nat. Rev. Endocrinol.* **18**, 219–229 (2022). 34983969 10.1038/s41574-021-00613-y

[4] Saeedi, P. et al. Global and regional diabetes prevalence estimates for 2019 and projections for 2030 and 2045: results from the International Diabetes Federation Diabetes Atlas, 9th edition. *Diabetes Res. Clin. Pract.* **157**, 107843 (2019). 31518657 10.1016/j.diabres.2019.107843

[5] Yazdanpanah, S. et al. Evaluation of glycated albumin (GA) and GA/HbA1c ratio for diagnosis of diabetes and glycemic control: a comprehensive review. *Crit. Rev. Clin. Lab. Sci.* **54**, 219–232 (2017). 28393586 10.1080/10408363.2017.1299684

[6] Weykamp, C. HbA1c: a review of analytical and clinical aspects. *Ann. Lab. Med.* **33**, 393–400 (2013). 24205486 10.3343/alm.2013.33.6.393 PMC3819436

[7] Day, A. HbA1c and diagnosis of diabetes. The test has finally come of age. *Ann. Clin. Biochem.* **49**, 7–8 (2012). 22218489 10.1258/acb.2011.011255

[8] Cohen, M. P. & Hud, E. Measurement of plasma glycoalbumin levels with a monoclonal antibody based ELISA. *J. Immunol. Methods* **122**, 279–283 (1989). 2794522 10.1016/0022-1759(89)90275-5

[9] Png, G. et al. Identifying causal serum protein–cardiometabolic trait relationships using whole genome sequencing. *Hum. Mol. Genet.* **32**, 1266–1275 (2023). 36349687 10.1093/hmg/ddac275 PMC10077504

[10] Dhindsa, R. S. et al. Rare variant associations with plasma protein levels in the UK Biobank. *Nature* **622**, 339–347 (2023). 37794183 10.1038/s41586-023-06547-x PMC10567546

[11] Zhao, J. H. et al. Genetics of circulating inflammatory proteins identifies drivers of immune-mediated disease risk and therapeutic targets. *Nat. Immunol.* **24**, 1540–1551 (2023). 37563310 10.1038/s41590-023-01588-w PMC10457199

[12] Sun, B. B. et al. Plasma proteomic associations with genetics and health in the UK Biobank. *Nature* **622**, 329–338 (2023). 37794186 10.1038/s41586-023-06592-6 PMC10567551

[13] Gilly, A. et al. Genome-wide meta-analysis of 92 cardiometabolic protein serum levels. *Mol. Metab.* **78**, 101810 (2023). 37778719 10.1016/j.molmet.2023.101810 PMC10582065

[14] Zhang, J. et al. Plasma proteome analyses in individuals of European and African ancestry identify *cis*-pQTLs and models for proteome-wide association studies. *Nat. Genet.* **54**, 593–602 (2022). 35501419 10.1038/s41588-022-01051-w PMC9236177

[15] Karakasilioti, I. et al. DNA damage triggers a chronic autoinflammatory response, leading to fat depletion in NER progeria. *Cell Metab.* **18**, 403–415 (2013). 24011075 10.1016/j.cmet.2013.08.011 PMC4116679

[16] Yu, Y. et al. Bioinformatics analysis of candidate genes and potential therapeutic drugs targeting adipose tissue in obesity. *Adipocyte* **11**, 1–10 (2022). 34964707 10.1080/21623945.2021.2013406 PMC8726706

[17] Elbein, S. C. et al. Global gene expression profiles of subcutaneous adipose and muscle from glucose-tolerant, insulin-sensitive, and insulin-resistant individuals matched for BMI. *Diabetes* **60**, 1019–1029 (2011). 21266331 10.2337/db10-1270 PMC3046820

[18] Tabasum, S. et al. EDIL3 as an angiogenic target of immune exclusion following checkpoint blockade. *Cancer Immunol. Res.* **11**, 1493–1507 (2023). 37728484 10.1158/2326-6066.CIR-23-0171 PMC10618652

[19] Gasca, J. et al. EDIL3 promotes epithelial–mesenchymal transition and paclitaxel resistance through its interaction with integrin α_V_β_3_ in cancer cells. *Cell Death Discov.* **6**, 86 (2020). 33014430 10.1038/s41420-020-00322-x PMC7494865

[20] Shen, W. et al. EDIL3 knockdown inhibits retinal angiogenesis through the induction of cell cycle arrest in vitro. *Mol. Med. Rep.* **16**, 4054–4060 (2017). 28765888 10.3892/mmr.2017.7122 PMC5646987

[21] Yu, C.-G. et al. Endothelial progenitor cells in diabetic microvascular complications: friends or foes? *Stem Cells Int.* **2016**, 1803989 (2016). 27313624 10.1155/2016/1803989 PMC4903148

[22] Tahergorabi, Z. & Khazaei, M. Imbalance of angiogenesis in diabetic complications: the mechanisms. *Int. J. Prev. Med.* **3**, 827–838 (2012). 23272281 10.4103/2008-7802.104853 PMC3530300

[23] Bocher, O. et al. Disentangling the consequences of type 2 diabetes on targeted metabolite profiles using causal inference and interaction QTL analyses. *PLoS Genet.* **20**, e1011346 (2024). 39625957 10.1371/journal.pgen.1011346 PMC11642953

[24] Mandla, R. et al. Multi-omics characterization of type 2 diabetes associated genetic variation. Preprint at *medRxiv*10.1101/2024.07.15.24310282 (2024).

[25] Peluso, C. et al. *TYK2* rs34536443 polymorphism is associated with a decreased susceptibility to endometriosis-related infertility. *Hum. Immunol.* **74**, 93–97 (2013). 23000200 10.1016/j.humimm.2012.09.007

[26] Fink-Baldauf, I. M., Stuart, W. D., Brewington, J. J., Guo, M. & Maeda, Y. CRISPRi links COVID-19 GWAS loci to LZTFL1 and RAVER1. *EBioMedicine* **75**, 103806 (2022). 34998241 10.1016/j.ebiom.2021.103806 PMC8731227

[27] Mahajan, A. et al. Multi-ancestry genetic study of type 2 diabetes highlights the power of diverse populations for discovery and translation. *Nat. Genet.* **54**, 560–572 (2022). 35551307 10.1038/s41588-022-01058-3 PMC9179018

[28] Vujkovic, M. et al. Discovery of 318 new risk loci for type 2 diabetes and related vascular outcomes among 1.4 million participants in a multi-ancestry meta-analysis. *Nat. Genet.* **52**, 680–691 (2020). 32541925 10.1038/s41588-020-0637-y PMC7343592

[29] Suzuki, K. et al. Genetic drivers of heterogeneity in type 2 diabetes pathophysiology. *Nature* **627**, 347–357 (2024). 38374256 10.1038/s41586-024-07019-6 PMC10937372

[30] Barrett, J. C. et al. Genome-wide association study and meta-analysis find that over 40 loci affect risk of type 1 diabetes. *Nat. Genet.* **41**, 703–707 (2009). 19430480 10.1038/ng.381 PMC2889014

[31] Batista, J. P., Faria, A. O., Ribeiro, T. F. & Simões e Silva, A. C. The role of renin–angiotensin system in diabetic cardiomyopathy: a narrative review. *Life* **13**, 1598 (2023). 37511973 10.3390/life13071598 PMC10381689

[32] Abuissa, H., Jones, P. G., Marso, S. P. & O’Keefe, J. H. Angiotensin-converting enzyme inhibitors or angiotensin receptor blockers for prevention of type 2 diabetes: a meta-analysis of randomized clinical trials. *J. Am. Coll. Cardiol.* **46**, 821–826 (2005). 16139131 10.1016/j.jacc.2005.05.051

[33] Hall, K. T. et al. Catechol-O-methyltransferase association with hemoglobin A1c. *Metabolism* **65**, 961–967 (2016). 27282867 10.1016/j.metabol.2016.04.001 PMC4924514

[34] Breeyear, J. H. et al. Adaptive selection at G6PD and disparities in diabetes complications. *Nat. Med*. 2480–2488 (2024). 10.1038/s41591-024-03089-1 PMC11555759 38918629

[35] Wheeler, E. et al. Impact of common genetic determinants of Hemoglobin A1c on type 2 diabetes risk and diagnosis in ancestrally diverse populations: a transethnic genome-wide meta-analysis. *PLoS Med.* **14**, e1002383 (2017). 28898252 10.1371/journal.pmed.1002383 PMC5595282

[36] Picó, C., Palou, M., Pomar, C. A., Rodríguez, A. M. & Palou, A. Leptin as a key regulator of the adipose organ. *Rev. Endocr. Metab. Disord.* **23**, 13–30 (2022). 34523036 10.1007/s11154-021-09687-5 PMC8873071

[37] Andrade-Oliveira, V., Câmara, N. O. S. & Moraes-Vieira, P. M. Adipokines as drug targets in diabetes and underlying disturbances. *J. Diabetes Res.* **2015**, 681612 (2015). 25918733 10.1155/2015/681612 PMC4397001

[38] Shpakov, A. O. [The role of alterations in the brain signaling systems regulated by insulin, IGF-1 and leptin in the transition of impaired glucose tolerance to overt type 2 diabetes mellitus]. *Tsitologiia* **56**, 789–799 (2014). 25707205

[39] Barber, M. et al. Diabetes-induced neuroendocrine changes in rats: role of brain monoamines, insulin and leptin. *Brain Res.* **964**, 128–135 (2003). 12573521 10.1016/s0006-8993(02)04091-x

[40] Scott, C. P., Williams, D. A. & Crawford, D. L. The effect of genetic and environmental variation on metabolic gene expression. *Mol. Ecol.* **18**, 2832–2843 (2009). 19500250 10.1111/j.1365-294X.2009.04235.x PMC2705469

[41] Baltramonaityte, V. et al. A multivariate genome-wide association study of psycho-cardiometabolic multimorbidity. *PLoS Genet.* **19**, e1010508 (2023). 37390107 10.1371/journal.pgen.1010508 PMC10343069

[42] Richardson, T. G. et al. Evaluating the relationship between circulating lipoprotein lipids and apolipoproteins with risk of coronary heart disease: a multivariable Mendelian randomisation analysis. *PLoS Med.* **17**, e1003062 (2020). 32203549 10.1371/journal.pmed.1003062 PMC7089422

[43] Bonàs-Guarch, S. et al. Re-analysis of public genetic data reveals a rare X-chromosomal variant associated with type 2 diabetes. *Nat. Commun.* **9**, 321 (2018). 29358691 10.1038/s41467-017-02380-9 PMC5778074

[44] Kichaev, G. et al. Leveraging polygenic functional enrichment to improve GWAS power. *Am. J. Hum. Genet.* **104**, 65–75 (2019). 30595370 10.1016/j.ajhg.2018.11.008 PMC6323418

[45] Sakaue, S. et al. A cross-population atlas of genetic associations for 220 human phenotypes. *Nat. Genet.* **53**, 1415–1424 (2021). 34594039 10.1038/s41588-021-00931-x PMC12208603

[46] Hoffmann, T. J. et al. A large genome-wide association study of QT interval length utilizing electronic health records. *Genetics* **222**, iyac157 (2022). 36271874 10.1093/genetics/iyac157 PMC9713425

[47] Tabassum, R. et al. Genetic architecture of human plasma lipidome and its link to cardiovascular disease. *Nat. Commun.* **10**, 4329 (2019). 31551469 10.1038/s41467-019-11954-8 PMC6760179

[48] Choudhury, A. et al. Meta-analysis of sub-Saharan African studies provides insights into genetic architecture of lipid traits. *Nat. Commun.* **13**, 2578 (2022). 35546142 10.1038/s41467-022-30098-w PMC9095599

[49] Meex, R. C. R., Blaak, E. E. & van Loon, L. J. C. Lipotoxicity plays a key role in the development of both insulin resistance and muscle atrophy in patients with type 2 diabetes. *Obes. Rev.* **20**, 1205–1217 (2019). 31240819 10.1111/obr.12862 PMC6852205

[50] Dilworth, L., Facey, A. & Omoruyi, F. Diabetes mellitus and its metabolic complications: the role of adipose tissues. *Int. J. Mol. Sci.* **22**, 7644 (2021). 34299261 10.3390/ijms22147644 PMC8305176

[51] Gurdasani, D. et al. Uganda genome resource enables insights into population history and genomic discovery in Africa. *Cell* **179**, 984–1002 (2019). 31675503 10.1016/j.cell.2019.10.004 PMC7202134

[52] Fatumo, S. et al. Uganda Genome Resource: a rich research database for genomic studies of communicable and non-communicable diseases in Africa. *Cell Genom.* **2**, None (2022). 36388767 10.1016/j.xgen.2022.100209 PMC9646479

[53] Ritchie, M. E. et al. Limma powers differential expression analyses for RNA-sequencing and microarray studies. *Nucleic Acids Res.* **43**, e47 (2015). 25605792 10.1093/nar/gkv007 PMC4402510

[54] Benjamini, Y. & Hochberg, Y. Controlling the false discovery rate: a practical and powerful approach to multiple testing. *J. R. Stat. Soc. Ser. B Stat. Methodol.* **57**, 289–300 (1995).

[55] Yu, G., Wang, L.-G., Han, Y. & He, Q.-Y. clusterProfiler: an R package for comparing biological themes among gene clusters. *OMICS* **16**, 284–287 (2012). 22455463 10.1089/omi.2011.0118 PMC3339379

[56] Petrera, A. et al. Multiplatform approach for plasma proteomics: complementarity of olink proximity extension assay technology to mass spectrometry-based protein profiling. *J. Proteome Res.* **20**, 751–762 (2021). 33253581 10.1021/acs.jproteome.0c00641

[57] Davis, J. R. et al. An efficient multiple-testing adjustment for eQTL studies that accounts for linkage disequilibrium between variants. *Am. J. Hum. Genet.* **98**, 216–224 (2016). 26749306 10.1016/j.ajhg.2015.11.021 PMC4716687

[58] Chang, C. C. et al. Second-generation PLINK: rising to the challenge of larger and richer datasets. *Gigascience* **4**, 7 (2015). 25722852 10.1186/s13742-015-0047-8 PMC4342193

[59] Cinar, O. & Viechtbauer, W. The poolr package for combining independent and dependent p values. *J. Stat. Softw.* **101**, 1–42 (2022).

[60] Giambartolomei, C. et al. Bayesian test for colocalisation between pairs of genetic association studies using summary statistics. *PLoS Genet.* **10**, e1004383 (2014). 24830394 10.1371/journal.pgen.1004383 PMC4022491

[61] Hemani, G. et al. The MR-Base platform supports systematic causal inference across the human phenome. *eLife* **7**, e34408 (2018). 29846171 10.7554/eLife.34408 PMC5976434

[62] Tian, D. et al. GWAS Atlas: a curated resource of genome-wide variant-trait associations in plants and animals. *Nucleic Acids Res.* **48**, D927–D932 (2020). 31566222 10.1093/nar/gkz828 PMC6943065

